# An Artificial Intelligence Approach to Support Detection of Neonatal Adverse Drug Reactions Based on Severity and Probability Scores: A New Risk Score as Web-Tool

**DOI:** 10.3390/children9121826

**Published:** 2022-11-26

**Authors:** Nadir Yalçın, Merve Kaşıkcı, Hasan Tolga Çelik, Karel Allegaert, Kutay Demirkan, Şule Yiğit, Murat Yurdakök

**Affiliations:** 1 Department of Clinical Pharmacy, Faculty of Pharmacy, Hacettepe University, Ankara 06230, Turkey; 2 Department of Biostatistics, Faculty of Medicine, Hacettepe University, Ankara 06230, Turkey; 3 Division of Neonatology, Department of Child Health and Diseases, Faculty of Medicine, Hacettepe University, Ankara 06230, Turkey; 4 Department of Pharmaceutical and Pharmacological Sciences, KU Leuven, 3000 Leuven, Belgium; 5 Department of Development and Regeneration, KU Leuven, 3000 Leuven, Belgium; 6 Department of Hospital Pharmacy, Erasmus Medical Center, 3015 GD Rotterdam, The Netherlands

**Keywords:** adverse drug reaction, machine learning, prediction, adverse event, newborn, risk analysis

## Abstract

Background: Critically ill neonates are at greater risk for adverse drug reactions (ADRs). The differentiation of ADRs from reactions associated with organ dysfunction/immaturity or genetic variability is difficult. Methods: In this prospective cohort study, each ADR was assessed using newborn-specific severity and probability scales by the clinical pharmacist. Subsequently, a machine learning-based risk score was designed to predict ADR presence in neonates. Results: In 98/412 (23.8%) of (56.3%; male) neonates included, 187 ADRs (0.42 ADR/patient) were determined related to 49 different drugs (37.12%). Drugs identified as high risk were enoxaparin, dexmedetomidine, vinblastine, dornase alfa, etoposide/carboplatin and prednisolone. The independent variables included in the risk score to predict ADR presence, according to the random forest importance criterion, were: systemic hormones (2 points), cardiovascular drugs (3 points), diseases of the circulatory system (1 point), nervous system drugs (1 point), and parenteral nutrition treatment (1 point), (cut-off value: 3 points). This risk score correctly classified 91.1% of the observations in the test set (c-index: 0.914). Conclusions: Using the high-performing risk score specific to neonates, it is expected that high-risk neonatal ADRs can be determined and prevented before they occur. Moreover, the awareness of clinicians of these drugs can be improved with this web-tool, and mitigation strategies (change of drug, dose, treatment duration, etc.) can be considered, based on a benefit-harm relationship for suspected drugs with a newborn-centered approach.

## 1. Introduction

It is known that the reported adverse drug reactions (ADRs) in neonates are more common and more severe than in other age groups. According to the double-center study examining potential ADRs for about ten thousand patients, neonatal intensive care unit (NICU) patients had a higher risk of having an ADRs [1]. In another study examining the risk factors for ADRs in children, the use of 4 or more neoplasm and circulatory system drugs significantly increased the prevalence of ADRs [2]. ADRs were most frequently reported by pharmacists (89%), nurses (10%), and physicians (1%). Although 93% of ADRs were reported to the physicians, only 29% of these ADRs were documented in the patient’s medical chart [3].

According to a study examining the risk factors for adverse events in neonates, it was determined that 22.5% of the suspected adverse reactions were drug-related. The most frequently identified risk factors (‘triggers’) for the presence of an ADR were decreased oxygen saturation, increased intestinal motility, vomiting, increased creatinine and blood urea nitrogen (BUN), non-steroidal anti-inflammatory drugs or caffeine-related necrotizing enterocolitis (NEC), flumazenil prescription, excessive sedation, or disturbances in electrolytes (hypercalcemia, hyperkalemia, or hypernatremia) [4].

With the high rate of use of off-label drugs in neonates, drug-related problems such as ADRs increase [5]. According to a pilot study examining the relationship between off-label drug use and MEs, at least one off-label drug is prescribed to 71% of patients, and it is known that these drugs increased MEs 20.4 times compared to drugs used in accordance with the indication [6].

In clinical practice, it is very difficult to determine whether undesirable and unexpected events seen in patients are drug-induced or not. For this reason, there are scales in the literature to detect and assess causality. Along the same line, additional tools are needed to assess its severity in neonates [7,8].

The ‘Du’ version is a further developed ADRs algorithm, specific for newborns and based on the Naranjo algorithm [9]. Being able to distinguish between ADRs and clinical symptoms is important to improve pharmacotherapy. In this context, 13 questions should be evaluated as yes/no/unknown by the clinician to determine the probability of ADRs in this scale [10]. According to the final score obtained, the relationship of the drug-related undesirable effect; categorized as ‘definite’, ‘probable’, ‘possible’, or ‘unlikely’ [5].

Using a Delphi consensus, a neonatal adverse event severity scale (NAESS) was recently published. According to this report, 35 typical and common neurological, cardiovascular, infectious, respiratory, gastrointestinal, and general neonatal adverse events are each categorized as ‘mild’, ‘moderate’, ‘severe’, ‘life-threatening’, or ‘death’, with a generic scale as default [8].

The primary aim of this study was to obtain objective risk categories by integrating the severity with NAESS and probability with the ‘Du’ADRs algorithm into the risk matrix analysis by the multidisciplinary team including the clinical pharmacist. The subsequent aim was to design a machine learning-based clinical decision support tool (risk score) that predicts whether or not these identified ADRs will occur.

## 2. Materials and Methods

### 2.1. Study Design and Population

This prospective cohort study randomized with 10-fold cross validation was conducted between February 2020 and June 2021 in a NICU with 22-bed capacity. All admitted neonates were considered, but neonates with preexisting hepatic or renal impairment were excluded from the study. Hacettepe University Institutional Review Board approved this study, and written informed consent was obtained from each participant’s parent/legal guardian (decision no. 2020/11-21). Additionally, this study was registered on the website ClinicalTrials.gov (NCT04899960).

### 2.2. Data Acquisition

Patients were followed up on a daily basis to assess their clinical status by a multidisciplinary team comprising physicians, nurses, and a clinical pharmacist. Each patient’s demographic, clinical, and adverse event data were obtained during routine follow-up. All patients were diagnosed using the International Classification of Diseases 10th Revision (ICD-10) codes, and drugs were classified using Anatomical Therapeutic Chemical (ATC) codes.

### 2.3. Causal Probability, Severity, and an ADR Risk Matrix

In this study, when a suspected ADR was detected, first the ‘Du’ ADRs algorithm was applied to determine the causality, and the NAESS was applied to determine the severity of the ADRs in NICU patients [7,8]. By multiplying the two scores obtained, the risk score of that ADR was obtained for each ADR that occurred in each neonate with a risk matrix analysis.

### 2.4. Random Forest Model Development, Optimization, and Validation

Before creating the model, the variables to be included in the model should be determined. For this reason, univariate analyses were performed and variables with a *p* value below 0.20 were determined. By evaluating the correlations between the variables and their clinical significance, the candidate variables to enter the model were determined. The recursive feature selection (RFE) method in the caret package was used to prevent bias in selecting the most significant ones among these variables. The RFE method used has 10-fold cross-validation and selects variables by taking into account the importance of variables in the random forest (RF) method. The importance orders of the independent variables that were decided to be included in the final model were obtained according to the RF for feature importance [11].

The model created for the prediction of presence of ADRs was decided to be a logistic regression model, due to the high-performance measurements. The data were randomly divided into train and test sets (70:30) using a 10-fold cross-validation method with R Program (caret package). Since the dependent variable, the presence of ADR, has an uneven distribution in the train set (ADRs vs. non-ADRs: 23% vs. 77%), a random sampling method was used in the train set to improve the performance of the model. For this reason, various methods were tried and finally the performance of the model was improved with a combination of over-sampling and under-sampling methods, the ovun.sample function in the ROSE package. The scientific and reliable method applied here is based on increasing the minority class by random sample selection while keeping the total number of observations in the train set, and decreasing the majority class. As a result, a more evenly distributed train set was obtained (ADRs vs. non-ADRs: 47% vs. 53%).

## 3. Results

### 3.1. Clinical Characteristics

During the study period, 468 neonates were admitted to a tertiary referral hospital’s 22-bed NICU. Fifty-six neonates were excluded due to non-survival without ADR (*n* = 21, 4.5%) or because they did not receive systemic medication treatment (*n* = 35, 7.4%). Consequently, 412 neonates were included in the study: 232 (56.3%) were males, 177 (43%) were born preterm (<37 weeks), and 172 (41.7%) had low birth weight (2500 g). The median (IQR) postnatal age (PNA) was 1 (1) day, and the median (IQR) length of hospital stay (LOS) was 8 (11) days.

A total of 412 NICU patients (5.53 drugs/patient/day) were studied prospectively, with 2280 medications prescribed in 32,925 patient days and 11,908 medication orders (28.9 orders/patient) prescribed using the computerized physician order entry (CPOE) system. Throughout the hospitalization period, the total number of drugs and anti-infectives used had median (range) values of 3 (0–29) and 2 (0–9), respectively. The most commonly prescribed medications were anti-infective (38.82%), alimentary tract and metabolism (32.89%), and nervous system (8.07%). During the study period, 131 various medications were prescribed. The most commonly used medications were intravenous fluids (12.06%), gentamicin (8.03%), and ampicillin (7.81%), respectively.

### 3.2. Characteristics of Suspected ADRs: Probability and Severity Scores

While performing the risk analysis to determine if the clinical or laboratory findings are related to the drug, the time interval when the drug was started and stopped, and the basal values (markers) observed in the patient before and after it was started were considered. A total of 187 ADRs (0.42 ADRs/patient) were detected from 49 (37.12%) different drugs in 98 (23.8%) neonates (Table 1). The mean (SD) number of ADRs was found to be 1.91 (1.32) in these patients (min-max: 1–6). In addition, one ADR in 52% of the patients, two ADRs in 28.6%, and more than two ADRs in 19.4% have been observed.

The drugs with the most ADRs were meropenem, dexamethasone, and vancomycin, respectively (Table 1). The medications with the least incidence of ADRs were intravenous fluid (0.36%) and gentamicin (0.54%). Of the medications with ADRs, 29 (59.19%) were administered intravenously, 17 (34.69%) orally, 2 (4.08%) by inhalation, and 1 (2.04%) subcutaneously. There were no ADR-related death or sequelae reported during the study period. The time of occurrence of ADRs after starting the treatment varies between 1 to 30 days. The most common ADRs were thrombocytopenia, hyper/hypoglycemia, and electrolyte imbalances.

As a result of the use of probability and severity scales in the risk matrix, 16 (32.65%) of the ADRs were low risk, 27 (55.10%) were moderate, and 6 (12.25%) were high risk. The medications identified as high risk were enoxaparin, dexmedetomidine, vinblastine, dornase alfa, etoposide/carboplatin and prednisolone (Table 1). When the probability and severity analysis of all detected ADRs were evaluated, it was seen that the most common probability score was ‘probable’ (3 points out of 4) (36.37%) and the most common severity score was ‘moderate’ (2 points out of 5) (54.55%) for ADRs, in line with New ADR and NAESS, respectively (Table 2).

### 3.3. Development and Optimization of a Model to Predict the Presence of ADRs

According to univariate analysis, the candidate variables to be included in the model to predict whether an ADR will occur in a patient are: gestational age, birth weight, diseases of the circulatory system, intubation, surgical procedure, parenteral nutrition (PN) treatment, endocrine system, nervous system and cardiovascular system drugs (*p* < 0.20). Among these variables, the independent variables decided to be included in the outcome model according to the RF algorithm were: endocrine system drugs, cardiovascular system drugs, disease of the circulatory system, nervous system drugs, and PN treatment.

However, due to the uneven distribution between patients with and without ADRs, a model could not be developed to predict the presence of ADRs using ML algorithms with the variables obtained. Instead, a NICU-specific ADR risk score was developed with a threshold value that can be used in clinical practice, based on these variables. This risk score correctly classified 91.1% of the observations in the test set, with good performance (accuracy 0.911, sensitivity 0.758, selectivity 0.967, positive predictive value (PPV) 0.893, negative predictive value (NPV) 0.917, F1 score 0.820, and area under the receiver operating characteristic curve (AUC) 0.862). A high-performance model requires an F1 score of 0.70 and above. As the selectivity and PPV of the created risk score are high, it is seen that the false positive rate (which is not actually seen but estimated to be an ADR) is low. In other words, it can be said that 89.3% of the patients who are predicted to have ADRs according to the risk score actually have ADRs. The risk score was found by rounding the regression coefficients (β) into integers. Before obtaining the risk score, some indices related to the prediction accuracy of the model were calculated. These indices were obtained using the validation function in the rms R package [12]. A total of 1000 bootstrap samples were used for validation.

The concordance index (c-index) is a generalization of the AUC and its interpretation is similar to the AUC. A value above 0.70 is indicative of adequate discrimination. The c-index for this model was found to be 0.914. In general, the indices obtained were determined within the acceptable limits specified in the literature.

**Figure 1:**
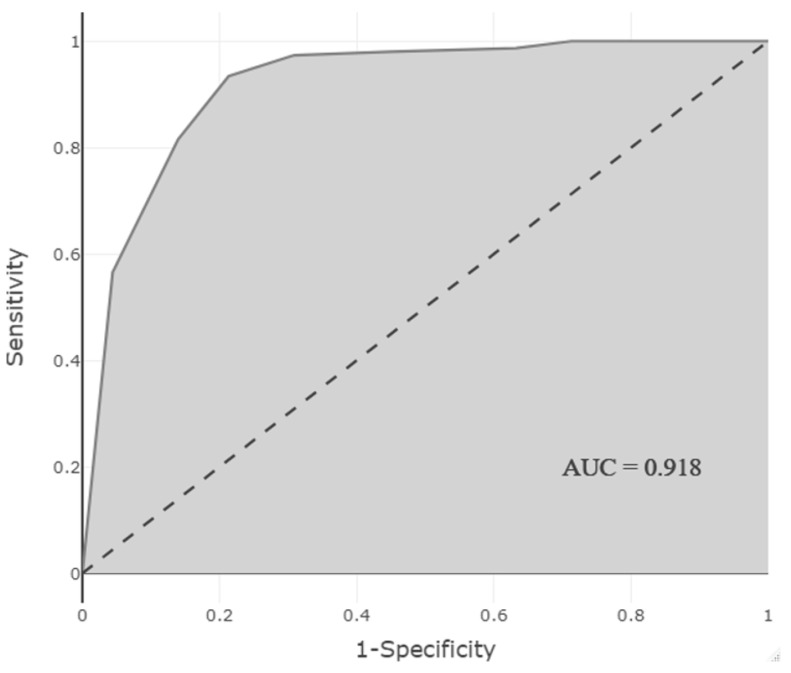
AUC-ROC curve showing the performance of the model predicting the presence of ADRs.

For this reason, the process of calculating the risk score was initiated. According to this risk score, ROC analysis obtained with the parameters of cardiovascular system (3 points), endocrine system (2 points), nervous system drugs (1 point), PN treatment (1 point), and diseases of the circulatory system (1 point), was easyROC [13] program (Table 3), and the AUC value for the risk score was found to be 0.918 (Figure 1). According to the Youden Index, the optimal cut-off value of this risk score was determined to be 3. After the risk score was taken, the performance of the test set was assessed. The risk score was calculated for the observations in the test set. While patients with a risk score of ≥3 points were observed to have ADRs, no ADRs were observed in patients with a risk score of <3 points. When compared with the real conditions of the patients, the performance criteria were similar to the train set, in which the cut-off value was determined.

Using the data of all patients, a significant positive correlation was obtained between the new ADRs risk score obtained by logistic regression and the mean risk correlation coefficient per ADR obtained from the risk matrix (Spearman rho = 0.657, *p* < 0.001). This shows that the mean risk per ADR and the new risk score were compatible with each other, and that the new ADR risk score obtained can be used reliably in clinical practice.

The parameters in the risk score obtained were detailed with a checklist that includes all cardiovascular, endocrine and nervous system drugs used in the NICU with a web-tool (http://softmed.hacettepe.edu.tr/NEO-DEER_Adverse_Effect/) (accessed on 22 November 2022). Thus, a practical ADR risk score that can be easily used in an online platform in the clinic has been brought to the literature and made available to clinicians.

## 4. Discussion

Neonates are vulnerable to ADRs, but these events are relatively infrequently reported in the literature. In our study, at least one ADR was observed in 23.8% of the patients during the hospitalization period. It was seen that 37.4% of the drugs prescribed cause ADRs. It is estimated that the number of ADRs seen per patient varies between 1–6, depending on the LOS, the number of comorbidities, and the number of drugs used. It was determined that the majority (59.49%) of drugs causing ADRs were administered intravenously. Intravenous drug use in NICUs is defined as a high-risk process. In a multicenter study conducted by NICU, it was found that 33% of 69 different intravenous drugs used in NICUs were not approved for neonates, 38% were high-risk, and only 63.5% were prepared and administered in standardized concentrations [14]. In our study, it is estimated that the administration of intravenous high-risk drugs may have an effect on the incidence of ADRs.

In a study comparing meropenem and imipenem/cilastatin in terms of the incidence of ADRs in hospitalized infants, it was found that more ADRs were seen with meropenem (62.8/1000 infant days vs. 40.7/1000 infant days, *p* < 0.001), but these ADRs were more severe with imipenem/cilastatin [15]. For this reason, in our study, it was seen that meropenem is the right choice in the NICU, but the platelet, eosinophil and AST levels of the neonates should be followed closely for the first 4 days from the start of meropenem. However, some of these ADRs are known to be preventable. According to a study that determined the incidence of preventable ADRs in the NICU, it was seen that anti-infectives were the most common cause [16]. In another study, it was determined that the drug causing the most ADRs in pediatric patients was vancomycin [3]. In addition to the increase in creatinine with the use of vancomycin, neutropenia was also observed in our study, especially in very low birth weight infants who may be frequently exposed to nephrotoxic drugs [17]. In our study, hyperglycemia was also determined in 13 patients associated with dexamethasone. Similarly to our study, Röhr et al. found that the risk of steroids-related hyperglycemia in very low birth weight infants increased 45 times [18]. However, when a risk analysis was performed for the three different steroids (dexamethasone, hydrocortisone, and methylprednisolone), it was determined that these ADRs were low or medium risk.

In our study, 94.21% of the ADRs and 87.75% of the drugs were found to be low or medium risk. Similarly, the rate of low-risk ADRs was found to be 89% [3]. Considering the high-risk ADRs, significant thrombocytopenia was found in two patients and microvascular hemorrhage in one patient with enoxaparin. Since the pharmacokinetics of preterm infants vary due to maturational differences, their therapeutic concentrations may also be variable. According to an 8-year retrospective study evaluating the use of enoxaparin in the NICU, minor gastrointestinal bleeding was found in three (18.8%) patients [19]. In our study, microvascular hemorrhage was observed in two (16.7%) patients treated with enoxaparin on the mean 17th day of treatment. In order to minimize such high-risk ADRs, it is recommended that the patient be monitored at the beginning of treatment, once a week, and every 3–4 weeks when the patient is stable, especially in preterm infants or patients with renal failure [20]. High-risk hypotension and a decrease in seizure threshold were detected in one patient each with dexmedetomidine. One patient had erythropenia and leukopenia, categorized as high-risk due to vinblastine use. In a patient who was administered dornase alfa for persistent atelectasis, drug-related respiratory obstruction was observed. One patient had severe neutropenia associated with etoposide. The last ADR, which was determined as high-risk, was an increase in INR as a result of prednisolone exposure, despite the fact that the patient is administered vitamin K once a week. The fact that these high-risk ADRs determined in the NICU also occur in drugs that are rarely seen shows the importance of monitoring in drugs that are not used routinely.

According to the risk score, endocrine system drugs (2 points), cardiovascular system drugs (3 points), circulatory system diseases (1 point), nervous system drugs (1 point), and PN treatment (1 point) were determined as risk factors for the presence of ADRs. Since cardiovascular drugs are generally started in neonates with circulatory system diseases, attention should be paid especially to ADRs in this population. Sugioka et al. determined that diseases of the circulatory system (OR: 3.94; *p* = 0.023) were a risk factor, increasing the incidence of ADRs [2]. Similarly, in our study, diseases of the circulatory system increased the risk of ADRs (OR: 3.87; *p* = 0.023). In our study, the risk score obtained with the risk factors was chosen randomly, and the absence of ADRs in all patients in the test set with a score below 3 indicates that the risk score was high performance. However, in order for this risk score to become an effective clinical tool, it should be integrated with the CPOE system, clinical pharmacy practices, or barcode scanning at the bedside. However, long-term outcomes and biochemical parameters will require confirmation of these risks. Risk analysis can be used in clinical practice as a complementary tool for this risk score. When the probabilities of the ADRs were examined in our study, it was determined that 69.53% of them were probable or definite. When the severity was examined, it was observed that 30.48% of them were severe or life-threatening ADRs. In a study in which three hundred and five ADRs were evaluated similarly in terms of probability and severity, while there were fewer probable or definite ADRs than our study, there were more severe or life-threatening ADRs than our study (50.12% and 40.7%, respectively) [21]. The high probability but low severity indicates that the ADRs determined may be of relatively low risk.

There are 76 different studies in the literature, 74% of which have emerged in the last 5 years, in which artificial intelligence is used to prevent and detect ADRs from drug discovery to pharmacovigilance in the general population. It was observed that 18% of these studies detected cardiovascular or renal ADRs, which were also frequently observed in our study [22,23]. McMaster et al. developed a machine learning algorithm that automatically detects 44.5% of ADRs reported by diagnosis code in predominantly adult patients (AUC: 0.803) [21]. The novelty of this paper is that we applied such an artificial intelligence approach in a NICU setting. The risk score obtained in our study correctly classified all patients without ADRs (AUC: 0.918). This result is expected to improve pharmacotherapy in neonates without causing alarm fatigue in clinicians with the use of a high-performance risk score in clinical practice to predict the presence of ADRs.

## 5. Conclusions

Although there are similar studies in the current literature, there is no high-performance risk score as a web-tool specific to neonates to assess ADR presence and severity. With the use of the obtained risk score in clinical practice, it is expected that patients with a high risk of ADRs will be identified and that ADRs will be prevented before they occur. Additionally, awareness of clinicians of these drugs can be improved with this web-tool (http://softmed.hacettepe.edu.tr/NEO-DEER_Adverse_Effect/) (accessed on 22 November 2022), and mitigation strategies (change of drug, dose, treatment duration, etc.) can be considered based on a benefit-harm relationship for suspected drugs with a newborn-centered approach.

## Figures and Tables

**Table 1:** The route of administration, onset day, type of ADRs, probability, severity, risk score, and risk category of the drugs (*n* = 187).

Drugs	Number of ADRs (Incidence %)	Route	Onset Day (Mean)	Type of ADRs	Probability Median (Min–Max)	Severity Median (Min–Max)	Risk Score Median (Min–Max)	Risk Category Median (Min–Max)
Meropenem	18 (16.07)	IV	4.38	Thrombocytopenia (12) Eosinophilia (4) Thrombocytosis (1) AST increase (1)	2 (1–4)	2 (1–3)	4 (2–8)	1 (1–2)
Dexamethasone	16 (57.14)	IV	2.68	Hyperglycemia (13) Hypertension (2) AST, Cr, BUN increase (1)	4 (1–4)	2 (1–4)	8 (2–12)	2 (1–3)
Vancomycin	15 (13.04)	IV	7.46	Neutropenia (11) Cr increase (4)	3 (1–4)	2 (1–4)	6 (1–12)	2 (1–3)
Furosemide	10 (38.46)	IV	2.62	Hypochloremia (6) Hypomagnesemia (1) Hyponatremia (1) Hypokalemia (1) Alkalosis (1)	4 (3–4)	2 (1–3)	8 (4–12)	2 (1–3)
PN	10 (6.32)	IV	15.50	Cholestasis (4) Hyperglycemia (2) TPNoma (1) Thrombus (1) Hyperkalemia (1) Hypernatremia (1)	3 (2–4)	2 (1–4)	8 (2–16)	2 (1–3)
Alprostadil	9 (47.36)	IV	5.22	Pyloric stenosis (8) Hypotension (1)	2 (2–3)	3 (2–3)	6 (6–9)	2 (2–2)
Hydrocortisone	7 (46.66)	IV	2.57	Hyperglycemia (7)	3 (2–4)	2 (2–3)	6 (4–12)	2 (1–3)
Hydrochlorothiazide	5 (45.45)	Oral	6.60	Hypochloremia (2) Hyponatremia (1) Hypokalemia (1) Hyperglycemia (1)	3 (3–3)	2 (1–2)	6 (3–6)	2 (1–2)
Ibuprofen	5 (55.55)	Oral	2.80	Thrombocytopenia (5)	3 (2–4)	2 (2–3)	6 (6–8)	2 (2–2)
Allopurinol	4 (36.36)	Oral	3.00	Hypouricemia (2) INR increase (1) BUN increase (1)	3 (2–4)	1 (1–3)	3 (2–12)	1 (1–3)
Amikacin	4 (3.36)	IV	13.00	Cr increase (3) ALP increase (1)	3 (2–4)	2 (2–3)	6 (4–12)	2 (1–3)
Amiodarone	4 (100)	IV	8.50	TSH increase (2) Hemolytic anemia (1) Eosinophilia (1)	3 (3–4)	2 (1–3)	6 (3–9)	2 (1–2)
Fentanyl	4 (8.69)	IV	3.66	Tachycardia (2) Hypoactivity (1) Hypotension (1)	3 (2–4)	2 (1–3)	4 (3–12)	1 (1–3)
Propranolol	4 (44.44)	Oral	5.00	Hypoglycemia (3) Bradycardia (1)	2 (2–4)	2 (1–2)	4 (2–8)	1 (1–2)
Ciprofloxacin	4 (33.33)	IV	15.00	AST increase (3) Hyperalgesia (1)	2 (2–4)	2 (2–2)	4 (4–8)	1 (1–2)
Biotin	3 (100)	Oral	4.00	Pseudohyperthyroidism (2) Vomiting (1)	4 (4–4)	1 (1–2)	4 (4–8)	1 (1–2)
Enoxaparin	3 (25.00)	SC	17.00	Thrombocytopenia (2) Microvascular hemorrhage (1)	4 (4–4)	3 (2–3)	12 (8–12)	3 (2–3)
Fluconazole	3 (2.70)	IV	7.00	AST increase (3)	2 (2–3)	2 (2–2)	4 (4–6)	1 (1–2)
Methylprednisolone	3 (75.00)	IV	1.66	Hyperglycemia (3)	1 (1–2)	1 (1–2)	1 (1–4)	1 (1–1)
Midazolam	3 (10.00)	IV	5.00	AST increase (1) Hypotension (1) Methemoglobinemia (1)	2 (2–4)	2 (1–2)	4 (2–8)	1 (1–2)
Morphine	3 (33.33)	IV	2.00	Seizure (1) Hypotension (1) Globe vesicle (1)	3 (2–4)	2 (2–3)	6 (6–8)	2 (2–2)
Octreotide	3 (100)	IV	8.33	Hyperglycemia (3)	3 (2–4)	2 (1–2)	6 (2–8)	2 (1–2)
Dexmedetomidine	2 (3.38)	IV	4.00	Hypotension (1) Seizure threshold descrease (1)	4 (4–4)	3 (3–3)	12 (12–12)	3 (3–3)
Phenobarbital	2 (9.09)	Oral	10.50	GGT increase (1) ALT increase (1)	3 (3–3)	2 (2–2)	6 (2–2)	2 (2–2)
Levetiracetam	2 (20.00)	Oral	10.00	Ocular deviation (1) GGT increase (1)	3	3	9	2
Milrinone	2 (13.33)	IV	13.50	Hypotension (2)	4	2	8	2
Vinblastine	2 (100)	IV	3.00	Leukopenia (1) Erythrocyte reduction (1)	4	3	12	3
Vitamin A	2 (8.69)	Oral	12.50	Thrombocytosis (1)	4	2	8	2
Diazoxide	1 (12.50)	Oral	7	Hyperbilirubinemia (1)	3	3	9	2
Dornaz alpha	1 (20.00)	İnhaler	4	Thrombocytopenia (1)	4	4	16	3
Etoposide/Carboplatin	1 (100)	IV	3	Airway obstruction (1)	4	4	16	3
Phenytoin	1 (25.00)	IV	2	Neutropenia (1)	3	1	3	1
Flecainide	1 (50.00)	Oral	1	AST increase (1)	3	3	9	2
Gentamicin	1 (0.54)	IV	3	Tachycardia (1)	2	2	4	1
Captopril	1 (14.28)	Oral	25	Cr increase (1)	3	2	6	2
Levosimendan	1 (33.33)	IV	2	Hypotension (1)	4	2	8	2
Maflor	1 (3.33)	Oral	8	Hypotension (1)	3	2	6	2
Metronidazole	1 (7.69)	IV	4	ALP increase (1)	2	2	4	1
Paracetamol	1 (25.00)	IV	1	AST increase (1)	3	1	3	1
Prednisolone	1 (100)	Oral	5	AST increase (1)	3	4	12	3
Salbutamol	1 (3.84)	İnhaler	8	INR increase (1)	3	2	6	2
Ampicillin + Sulbactam	1 (25.00)	IV	17	Hypokalemia (1)	3	2	6	2
Ceftriaxone	1 (100)	IV	2	ALP increase (1)	2	2	4	1
Sholl Solution	1 (50.00)	Oral	12	Hyperbilirubinemia (1)	4	2	8	2
Sotalol	1 (100)	Oral	5	Vomiting (1)	4	2	8	2
Spirinolactone	1 (16.66)	Oral	30	Hypoglycemia (1)	3	2	6	2
Terlipressin	1 (33.33)	IV	4	Gynecomastia (1)	4	2	8	2
Total Fluid	1 (0.36)	IV	1	Hyponatremia (1)	3	1	3	1
Ursodiol	1 (50.00)	Oral	8	Hyperglycemia (1)	2	2	4	1

Probability: New ADRs Algorithm, severity: NAESS, green: low risk, yellow: moderate risk, red: high risk, IV: intravenous, SC: subcutaneous, Cr: Creatinine, GGT: Gamma glutamyl transferase, ALT: Alanine transaminase, AST: Aspartate transaminase, INR: Prothrombin time, BUN: Blood urea nitrogen, ALP: Alkaline phosphatase, PN: Parenteral nutrition, TSH: Thyroid stimulating hormone.

**Table 2:** Distribution of observed ADRs according to the probability and severity analysis.

		SEVERITY				
		*Mild (1)* *n = 28 (14.97%)*	*Moderate (2)* *n = 102 (54.55%)*	*Severe (3)* *n = 47 (25.13%)*	*Life Threatening (4)* *n = 10 (5.35%)*	*Death (5)* *-*
**PROBABILITY**	*Definite (4)* *n = 62 (33.16%)*	4	8	12	16	20
	*Probable (3)* *n = 68 (36.37%)*	3	6	9	12	15
	*Possible (2)* *n = 52 (27.80%)*	2	4	6	8	10
	*Unlikely (1)* *n = 5 (2.67%)*	1	2	3	4	5

Green: low risk, yellow: moderate risk, red: high risk.

**Table 3:** Regression analysis of the variables used to predict the presence of ADRs.

Variables	β	SE(β)	*p* *	OR	95% CI for OR	Risk Score
Endocrine system drugs	2.443	0.522	<0.001	11.508	4.134–32.039	2 points
Cardiovascular system drugs	2.702	0.501	<0.001	14.902	5.583–39.774	3 points
Diseases of the circulatory system	1.354	0.596	0.023	3.872	1.203–12.460	1 point
Nervous system drugs	1.026	0.394	0.009	2.790	1.288–6.042	1 point
Parenteral nutrition treatment	1.344	0.402	0.001	3.835	1.745–8.431	1 point

* p < 0.05 was considered statistically significant. OR: Odds ratio, CI: Confidence interval.

## Data Availability

The data presented in this study are available on request from the corresponding author. The data are not publicly available due to privacy and ethical restrictions.
